# Depth and direction effects in the prediction of static and shifted reaching goals from kinematics

**DOI:** 10.1038/s41598-023-40127-3

**Published:** 2023-08-12

**Authors:** A. Bosco, M. Filippini, D. Borra, E. A. Kirchner, P. Fattori

**Affiliations:** 1 Department of Biomedical and Neuromotor Sciences, University of Bologna, Bologna, Italy; 2 Alma Mater Research Institute for Human-Centered Artificial Intelligence, University of Bologna, Bologna, Italy; 3 Department of Electrical, Electronic and Information Engineering, University of Bologna, Bologna, Italy; 4 Department of Electrical Engineering and Information Technology, University of Duisburg-Essen, Duisburg, Germany; 5 Robotics Innovation Center, German Research Center for Artificial Intelligence GmbH, Kaiserslautern, Germany

**Keywords:** Neuroscience, Cognitive neuroscience, Computational neuroscience, Motor control

## Abstract

The kinematic parameters of reach-to-grasp movements are modulated by action intentions. However, when an unexpected change in visual target goal during reaching execution occurs, it is still unknown whether the action intention changes with target goal modification and which is the temporal structure of the target goal prediction. We recorded the kinematics of the pointing finger and wrist during the execution of reaching movements in 23 naïve volunteers where the targets could be located at different directions and depths with respect to the body. During the movement execution, the targets could remain static for the entire duration of movement or shifted, with different timings, to another position. We performed temporal decoding of the final goals and of the intermediate trajectory from the past kinematics exploiting a recurrent neural network. We observed a progressive increase of the classification performance from the onset to the end of movement in both horizontal and sagittal dimensions, as well as in decoding shifted targets. The classification accuracy in decoding horizontal targets was higher than the classification accuracy of sagittal targets. These results are useful for establishing how human and artificial agents could take advantage from the observed kinematics to optimize their cooperation in three-dimensional space.

## Introduction

Actions require complex cognitive processes that start from abstract action intentions and arrives to the description of motor mechanistic properties^1–3^. Typically, the interpretation and predictions about the actions and their intentions represent crucial aspects on which the human behaviour is based. Specifically, by a mechanistic perspective, the kinematic parameters of reach-to-grasp movements are modulated by the action intentions. In fact, the reach and the grasp components are affected by changes in both intrinsic and extrinsic features of the target and prior intentions^4,5^. The knowledge that the kinematics of human behaviour is modulated by different motor intentions opens to the possibility of using the observed kinematics as a cue to predict the action intentions. For example, Manera et al.^6^ showed the possibility to predict the agent’s intention to cooperate or compete with a partner by the observation of the initial reach and grasp phase of the entire movement and without contextual information. Similar results were found by Sartori et al.^7^ and another study by Cavallo et al.^8^ showed that the accuracy found in an intention prediction task was significantly related to a specific subset of kinematics features varying across different intentions. Another study showed that the similarity between the kinematics of agents and observers have a positive impact on the accuracy in recognition of intentions^9^. Furthermore, the use of an object influences the way we reach towards that object and grasp it^10,11^. In fact, what the actor intends to do with the object (e.g. throw a bottle, pour something from a bottle, or pass a bottle to another person) shapes the grasping kinematics for the same object^12^. And similarly, the actor’s social intentions such as cooperating with a partner, competing against an opponent, or performing an individual action also modulate the kinematics of reach-to-grasp movements^11,13^. Based on the evidence that the action execution is itself shaped by its final goal and/or by the physical properties of its target, the intention and outcome of an observed action can be accurately predicted. In fact, as the action is shaped by its final goal, the information about the goal is available to the observer well before the end of action execution. This can be applied to several ecological motor behaviours spanning from simple actions (e.g. reach-to-grasp an object) to more complex ones (e.g. playing an interactive ball game)^14^.

In the previously discussed studies, researchers investigated the possibility to predict action intentions or action goals from the kinematics of movement towards targets/objects that remained stable for the entire execution of the movement. However, it is still an open question the effect of an unexpected change in target goal position that occurs at different directions and depths with respect to the body during reaching execution on the prediction of the final goal. In addition, it is unknown the temporal structure of this prediction. In this study, we addressed these questions, and we characterized the information contained in the kinematics of reaching movement towards targets located at different directions and depths with respect to the body in a condition where the targets remained static for the entire duration of the movement and in a condition where the targets shifted to another position during the movement execution. To investigate the effect of reaching perturbation on the predictability of reaching goals and of intermediate positions (i.e., to quantify the amount of information of kinematics that enable the prediction of reaching), a recurrent neural network was used to decode the reaching goals over the trial course (i.e., over time) and the intermediate positions of reaching kinematics from the three-dimensional position of the pointing finger and the wrist of human participants.

## Results

**Figure 1:**
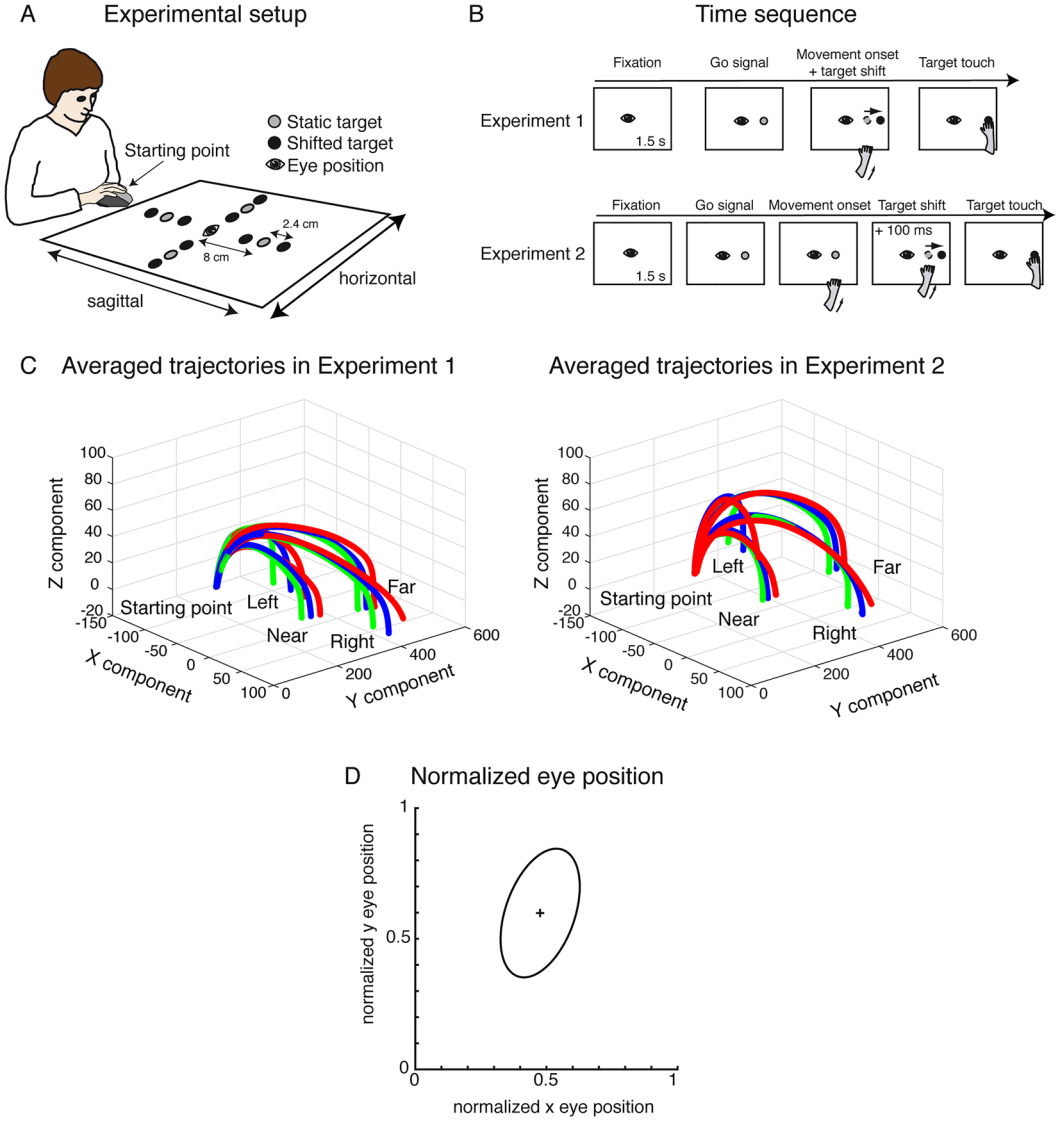
Experimental setup. (**A**) View of the target arrangement with respect to the participant’s body. The participants performed reaching movements with their right hand towards one of the 12 dots projected on the screen at different depths (near and far, sagittal dimension) and in different directions (left and right, horizontal dimension). Reaching movements were performed in a dimly lighted room, starting from the mouse located next to the body. Static targets are represented as grey dots, shifted targets are represented as black dots. (**B**) Time sequence of task. The eye symbol represents the fixation target; the filled grey dot represents the reaching target. The fixation target stayed on for 1.5 s and then the reaching target was turned on in one of the four static locations. As soon as the reaching target appeared on the screen, the participant had to reach with his/her right hand the position of the target while maintaining fixation on the fixation target. The fixation target lasted until the participant completed the movement. In Experiment 1 the target shift occurred at the movement onset, in Experiment 2 the target shift occurred 100ms after the movement onset (black dot). (**C**) Averaged reaching trajectories executed towards static and shifted targets located along direction and depth dimensions in Experiment 1 (left) and in Experiment 2 (right). Blue lines indicate trajectories towards static targets, red and green lines indicate trajectories towards shifted targets. (**D**) Example of individual normalized eye position reported in pixels displayed as the most likely ellipse surrounding 95% of eye data points during reaching. The black cross represents the center of the ellipse. 0.5 represents the center of the screen along the horizontal and sagittal dimension of the screen, 1 on the x axis represents the right limit of the screen, 1 on the y axis represents the furthest limit of the screen.

The 3D position of the index finger and the wrist was recorded in each participant during reaching movements performed towards 12 static and shifted spatial positions with 6 different direction angles and 6 different depth levels (see Fig. 1A–D for the experimental setup), covering a wide range of positions in the peripersonal space. Twelve participants executed the task with the target shifts at the onset of the movement (Experiment 1, see Fig. 1B) and 11 participants executed the task with the targets shift after 100 ms with respect to the movement onset (Experiment 2, see Fig. 1B). In both experiments, during each session, the sequence of presentation of static and shifted targets were randomized. At first, we analysed the movement kinematics by applying a computational approach based on sliding window decoding with a recurrent neural network (RNN), able to highlight the temporal evolution of the accuracy in predicting the final target goal for movements towards static and shifted targets from the recorded kinematics. This analysis was performed to evaluate the discriminability of movements from kinematics in the temporal domain. Then, with a similar approach as the previous one (based on a similar RNN and on sliding window decoding), we analyzed whether the reaching trajectories (and not only the reaching goal as before) can be predicted from past observations. This was performed to investigate whether also the prediction of intermediate positions was affected by the applied target shifts. Both analyses were conducted separately for horizontal and sagittal targets, and separately for different latencies in the application of the perturbation (i.e., the two experiments).

### Kinematic analysis of reaching movement corrections to targets along the horizontal and sagittal dimension

**Figure 2:**
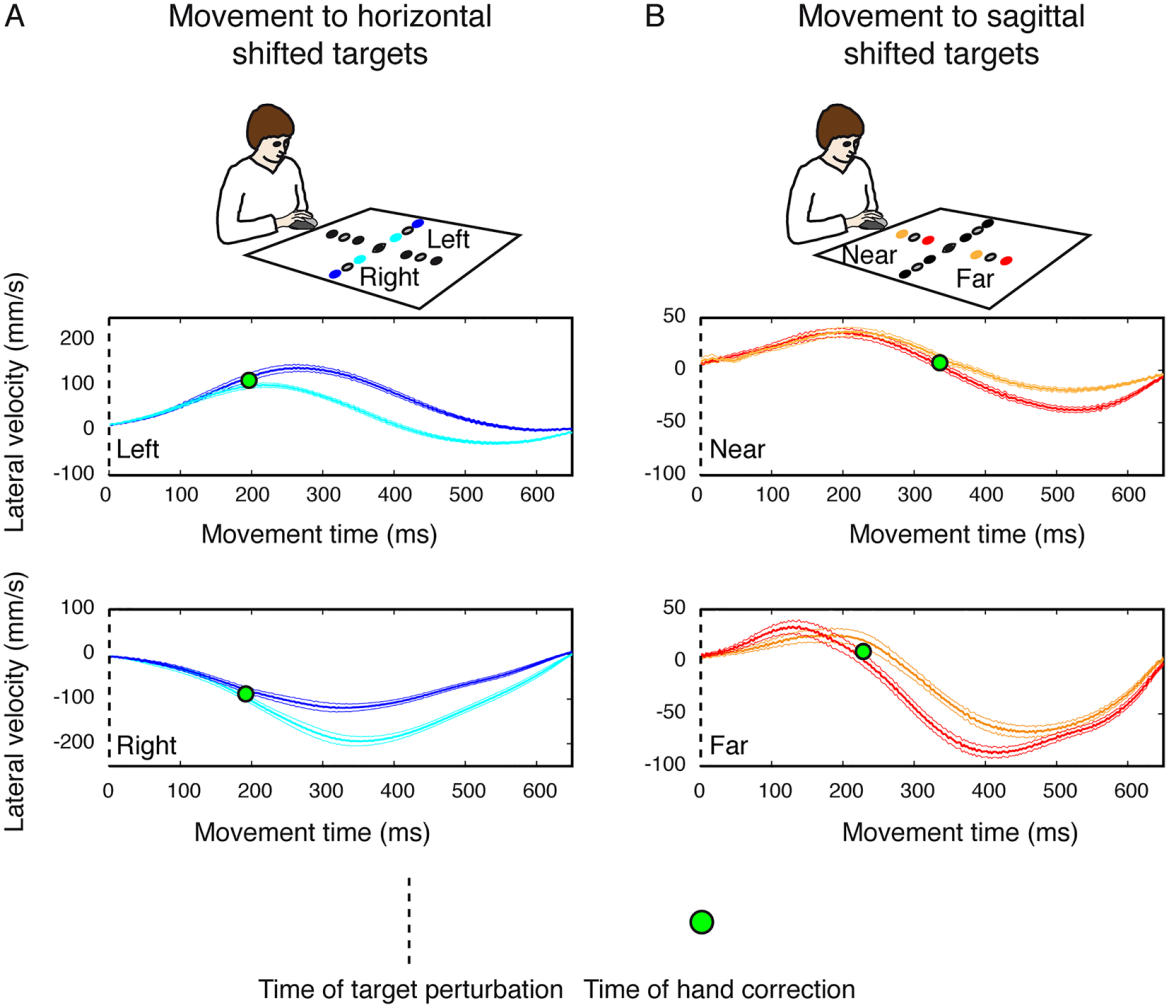
Average lateral velocity of the pointing finger during reaching to horizontal and sagittal shifted targets in Experiment 1. (**A**) Lateral velocity profiles corresponding to movements towards shifted targets to the left (top panel) and on the right (bottom panel). Positive values indicate deviation of movement to the right; negative values deviation of movement to the left. Green circle represents the time at which significant separation between the two traces occurs. Dashed vertical line correspond to the time of target shift. (**B**) Lateral velocity profiles corresponding to movements towards shifted near targets (top panel) and shifted far targets (bottom panel). Details as in (**A**).

To explore the dataset and to guide the subsequent analyses, we started with a kinematic analysis of reaching. As it is shown in the decoding analysis below, the kinematic properties were captured by the application of the Recurrent Neural Network decoder used to decode reaching target goals and movement trajectories. Specifically, we first performed a kinematic analysis on lateral velocity of reaching movements to evaluate the time at which the trajectories towards the shifted targets significantly deviated, causing the correction of the hand path originally directed to the static target. Figure 2 shows the averaged lateral velocity profiles as function of the movement time for shifted reaching actions in Experiment 1. To assess the time at which the hand correction occurred, we compared the velocities of movements to horizontal shifted targets evaluating the time of significant deviation by a two-tailed t-test (p < 0.05). For movement to horizontal left targets the deviation occurred after ~ 196 ms (Fig. 2A, upper panel) with respect to the target shift (onset of the movement, dotted vertical line in Fig. 2) and for movements to horizontal right targets after ~ 193 ms (Fig. 2A, lower panel) with respect to the target shift. For movements to sagittal near targets the correction occurred after ~ 357 ms (Fig. 2B, upper panel) and for movements to sagittal far targets after ~ 229 ms with respect the the target shift (Fig. 2B, lower panel). These results suggest that the hand corrections of movements towards the shifted sagittal targets occurred later than those of movements towards the shifted horizontal targets.

**Figure 3:**
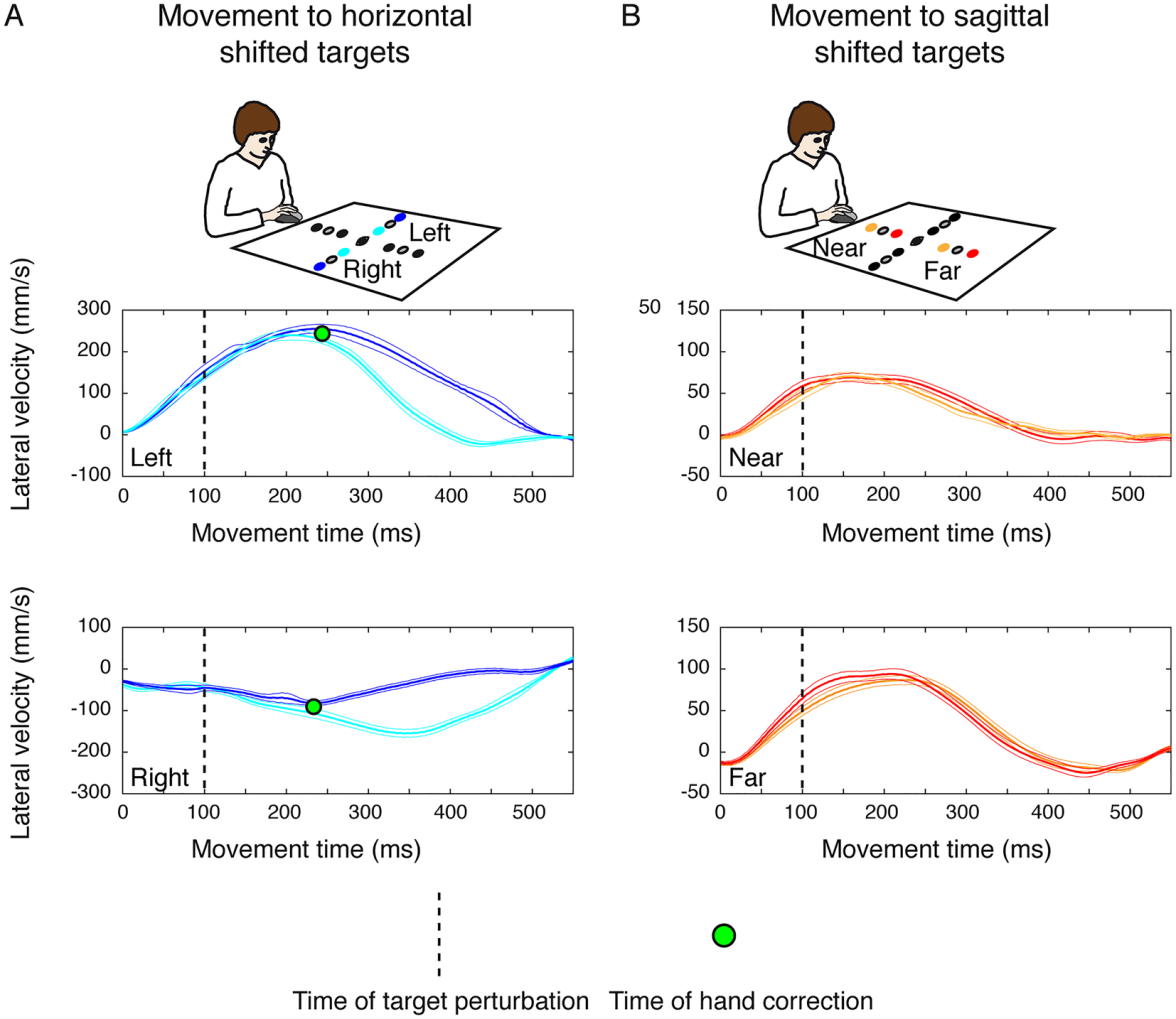
Average lateral velocity of the pointing finger during reaching to horizontal and sagittal shifted targets in Experiment 2. (**A**) Lateral velocity profiles corresponding to movements towards shifted targets on the left (top panel) and on the right (bottom panel). Positive values indicate deviation of movement to the right; negative values deviation of movement to the left. Green circle represents the time at which significant separation between the two traces occurs. Dotted vertical line correspond to the time of target shift. (**B**) Lateral velocity profiles corresponding to movements towards shifted near targets (top panel) and shifted far targets (bottom panel). Details as in (**A**).

**Figure 4:**
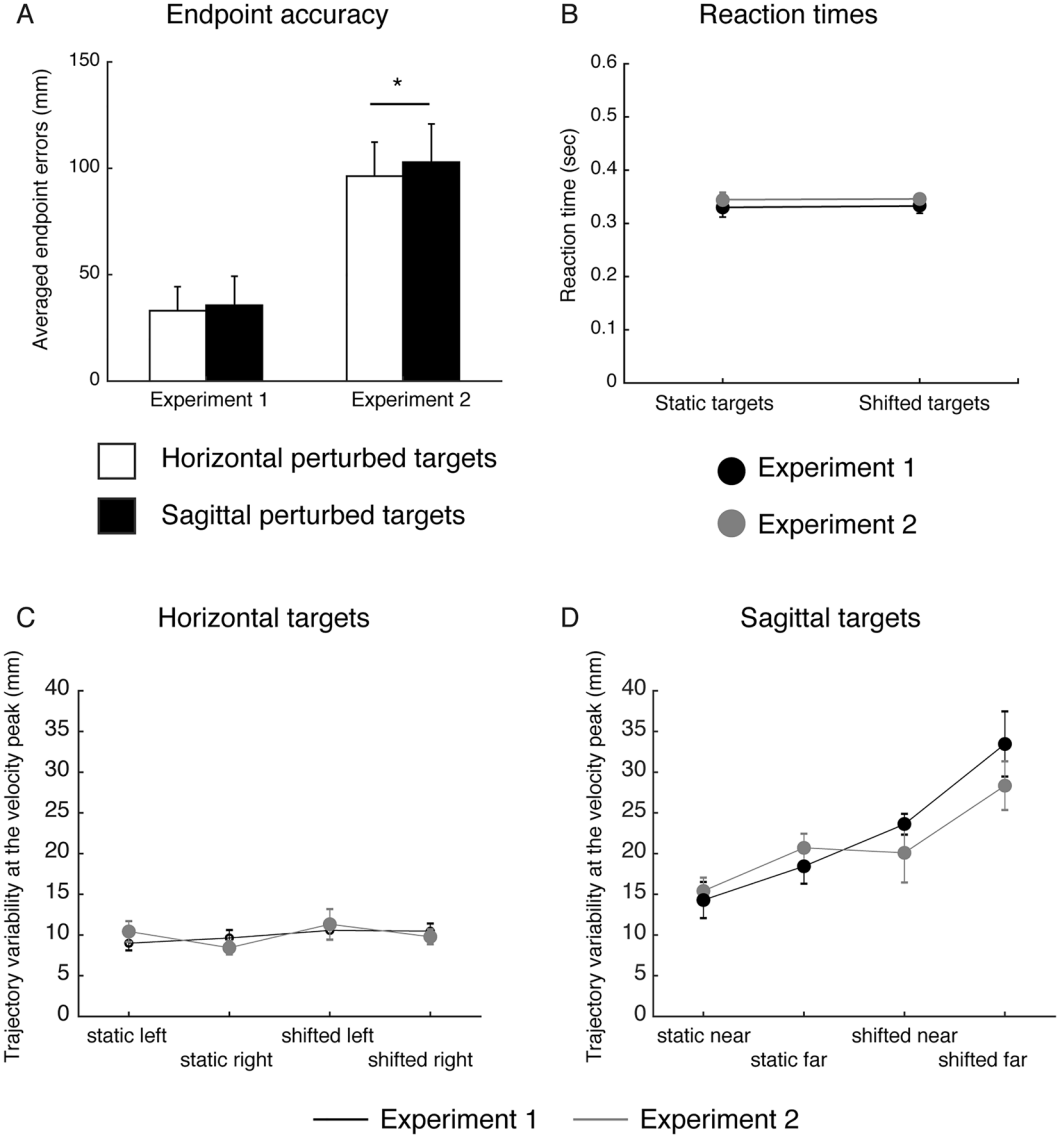
(**A**) Averaged reaching endpoints of Experiments 1 and 2. Averaged reaching endpoints calculated in the horizontal shifted targets (white columns) and sagittal shifted targets (black columns) in Experiments 1 and 2, respectively. Increased reaching errors are present in the sagittal dimension in both experiments, particularly in Experiment 2. (**B**) Averaged reaction times of reaching movement initiation of Experiments 1 (black dots) and 2 (grey dot) for static and shifted targets. (**C**) Distribution of trajectory variability in the X dimension for movements directed to static and shifted horizontal targets at the point of peak velocity of the movement in Experiment 1 (black dots) and Experiment 2 (grey dots). (**D**) Distribution of trajectory variability in the Y dimension for movements directed to static and shifted sagittal targets at the point of peak velocity of the movement in Experiment 1 (black dots) and Experiment 2 (grey dots).

The same kinematic analysis was performed for experiment 2. Figure 3 shows the averaged lateral velocity profiles as function of the movement time for shifted reaching actions in Experiment 2. In this case, we found that for movement towards horizontal left targets the deviation occurred after ~ 144 ms (Fig. 3A, upper panel) with respect to the target shift (100 ms after the movement onset, dotted vertical line in Fig. 3) and for movements to horizontal right targets after ~ 133 ms (Fig. 3A, lower panel) with respect to the target shift. In Experiment 2, we did not find significant corrections of the hand when the movements were performed towards shifted near and far targets (Fig. 3B, upper and lower panel). We hypotesized that the differences in the latency of target shift in the two experiments and the lack of hand correction had impact on the movement accuracy. Figure 4A shows the endpoint errors for movements performed to horizontal and sagittal shifted targets in the two experiments. It is evident that when the target shift was at the onset of the movement, the participants were able to successfully correct the hand and performed smaller endpoint errors compared to Experiment 2. No significant differences were found between endpoint errors towards horizontal and sagittal shifted targets (Fig. 4A, left, two-tailed t-test, p > 0.05). On the other hand, when the target shift occurred after 100 ms after the movement onset (Experiment 2), the participants performed larger errors in movements towards shifted horizontal and sagittal targets. The endpoint errors executed towards shifted sagittal targets were significantly larger than the endpoint errors executed towards shifted horizontal targets (Fig. 4A, right, two-tailed t-test, p > 0.05). These results are in line with the evidence that we did not find significant deviations in the lateral velocity of movements performed to sagittal targets in Experiment 2 suggesting that the lack of hand path correction leads to increased reaching errors. However, to exclude the possibility that the differences found between Experiment 1 and Experiment 2 were due to differences in motor plan and in reaching strategies of the two groups of participants, we calculated the reaction times of reaching movement initiation and the trajectory variability at the peak velocity, respectively. No significant differences were found in the comparison of reaction times for reaching movements to static and shifted among the two experiments suggesting that the same motor plan was used by the participants of Experiment 1 and Experiment 2 (two sample t-test, corrected for multiple comparisons, P > 0.025, see Fig. 4B). In Fig. 4C, D we reported the trajectory variability analysis to evaluate whether there were significant modifications of trajectory paths across static and shifted trials in the two experiments at the moment of peak of velocity that represents a relevant point during the reaching movement. Our hypothesis was that if the motor strategies were different in the execution of static and shifted trials among the two groups of participants, the distribution of trajectory variability at the peak of velocity should be statistically different between the two experiments. However, we found no significant differences in trajectory variabilities at the peak of velocity between movements towards static targets of Experiment 1 and Experiment 2 neither between movements towards shifted targets of Experiment 1 and Experiment 2 (two sample t-test, corrected for multiple comparisons, P > 0.0125; see Fig. 4C, D).

### Temporal evolution of static and shifted reaching goals classification

To study the temporal dynamics of discriminability of reaching endpoints, RNN-based classifiers were trained on sliding windows within the trial course. In the following analyses we investigated the temporal evolution of the accuracy in recognizing the static or shifted target positions along the horizontal and sagittal dimensions in Experiment 1 (target shift at the onset of the movement) and in Experiment 2 (target shift after 100 ms from movement onset). In general, we found that the time course of the accuracy (recognition rate) in predicting static and shifted targets shows similar trends in both horizontal and sagittal dimension and in the two Experiments. No significant differences were found between the time course of decoding accuracy of static targets between Experiment 1 and Experiment 2 suggesting that the two groups presented the same baseline (SPM1d with two-sample Hotellings’ T2 test, p > 0.05).

**Figure 5:**
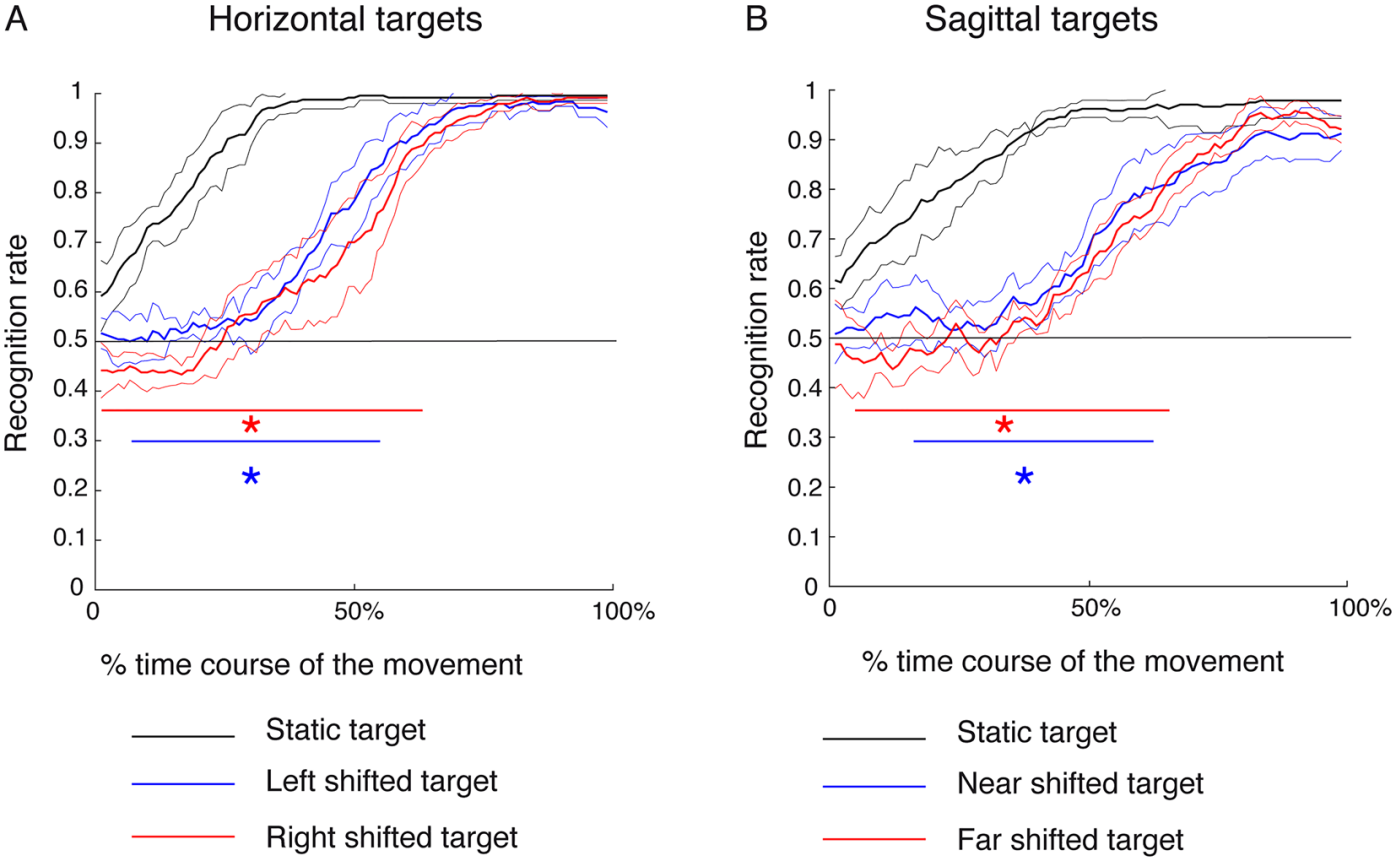
Averaged time course of the classification accuracy of movement goals in Experiment 1 as scored by RNN classifiers. (**A**) Average time course across subjects of classification accuracy of static (black line) and shifted horizontal targets (blue and red lines). Thin lines are standard deviations. The red and blue horizontal lines represent the intervals of significant separation of the two lines (blue and red lines) corresponding to the shifted targets accuracy with the line corresponding to static targets (black line) assessed by Hotelling’s test (p < 0.05). The horizontal black line represents the chance level (0.5 for the performed 2-class classifications). (**B**) Average time course across subjects of classification accuracy of static (black line) and shifted sagittal targets (blue and red lines). All details as in (**A**). It is evident that the prediction of shifted targets from the trajectories is slower than the prediction of static targets in both horizontal and sagittal dimensions.

In Experiment 1 when the target shift occurs at movement onset, the accuracy in the prediction of static horizontal targets was significantly higher than the one of left shifted targets (from 7 to 56% of movement execution, SPM1d with two-sample Hotellings’ T2 test, p < 0.05) and also than the one of right shifted targets (from 1 to 64% of movement execution, SPM1d with two-sample Hotellings’ T2 test, p < 0.05), see Fig. 5A. Furthermore, the accuracy in predicting the static sagittal targets was significantly higher than the one of near shifted targets (from 16 to 63% of movement execution, SPM1d with two-sample Hotellings’ T2 test, p < 0.05), and also than the one of far shifted targets (from 5 to 66% of movement execution, SPM1d with two-sample Hotellings’ T2 test, p < 0.05), see Fig. 5B. So, the RNN classifier reaches the maximum accuracy in predicting static targets earlier than in predicting shifted targets suggesting a significant effect of target shift on the RNN performance.

**Figure 6:**
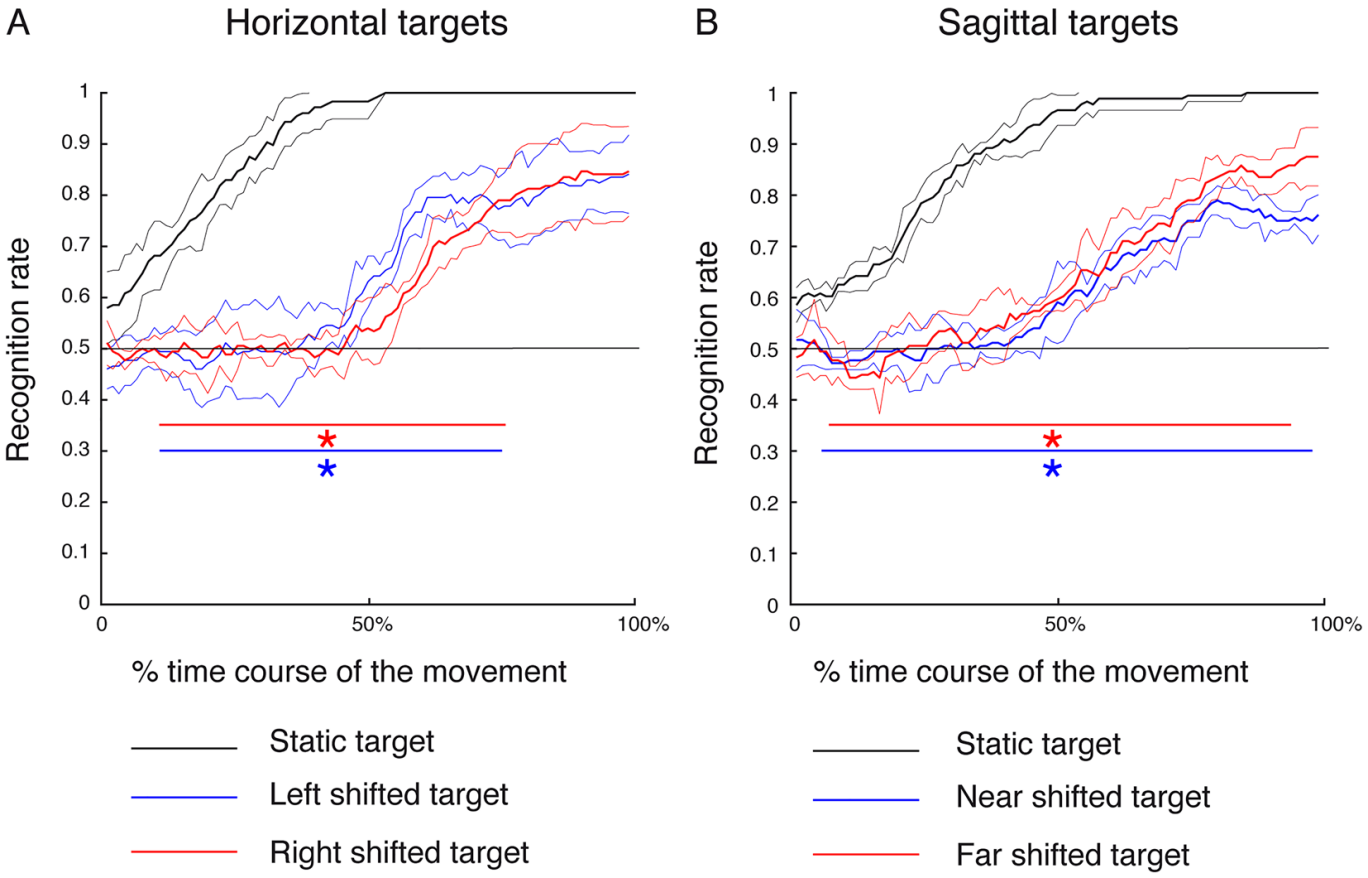
Averaged time course of the classification accuracy of movement goals in Experiment 2 as scored by RNN classifiers. (**A**) Horizontal targets. The horizontal solid line represents the chance level (0.5 for the performed 2-class classifications). (**B**) Vertical targets. All details as in Fig. 5. Differently from Experiment 1, in Experiment 2 we found that the prediction of shifted targets was less accurate in both horizontal and sagittal dimension.

In Experiment 2, when the target shift occurs 100 ms after movement onset, we found similar results as obtained from Experiment 1, but in wider intervals of movement execution compared to Experiment 1. Specifically, the accuracy in the prediction of horizontal static targets was significantly higher with respect to the one of shifted left targets from 11 to 76% of movement execution, and of shifted right targets from 11 to 77% of movement execution (SPM1d with two-sample Hotellings’ T2 test, p < 0.05), see Fig. 6A. Lastly, regarding the prediction of sagittal targets, the accuracy in predicting static sagittal targets was significantly higher with respect to those of near and far shifted targets (from 5.75 to 98.87% and from 7.24 to 94.89% of the movement execution, respectively, SPM1d with two-sample Hotellings’ T2 test, p < 0.05), see Fig. 6B. The results in Experiment 2 suggest significant effects of the target shift occurrence but also the timing of this shift because the prediction accuracy of shifted targets did not reach the maximum accuracy values as in Experiment 1. However, this phenomenon was not due to the fact that in Experiment 1 the classifier reached the maximum accuracy earlier having longer time interval between the target shift and the end of movement because the movement duration and the mean velocity calculated within this interval were not significantly different between Experiment 1 and Experiment 2 (two sample t-test, corrected for multiple comparisons, P > 0.0125). Moreover, to exclude the possibility that micro-movements before the onset of the reaching allowed some kind of predictability, we calculated the classification accuracy during the 500 ms before the movement onset. In both experiment and for all the reaching targets tested, the maximum classification accuracy was equal or below the chance level of 0.5. And finally, although the endpoint accuracy decreased in Experiment 2 (see Fig. 4A), the RNN classifier was able to recognize the target goals with a certain accuracy even in those movements with large endpoint errors despite a high variability (data not shown).

**Figure 7:**
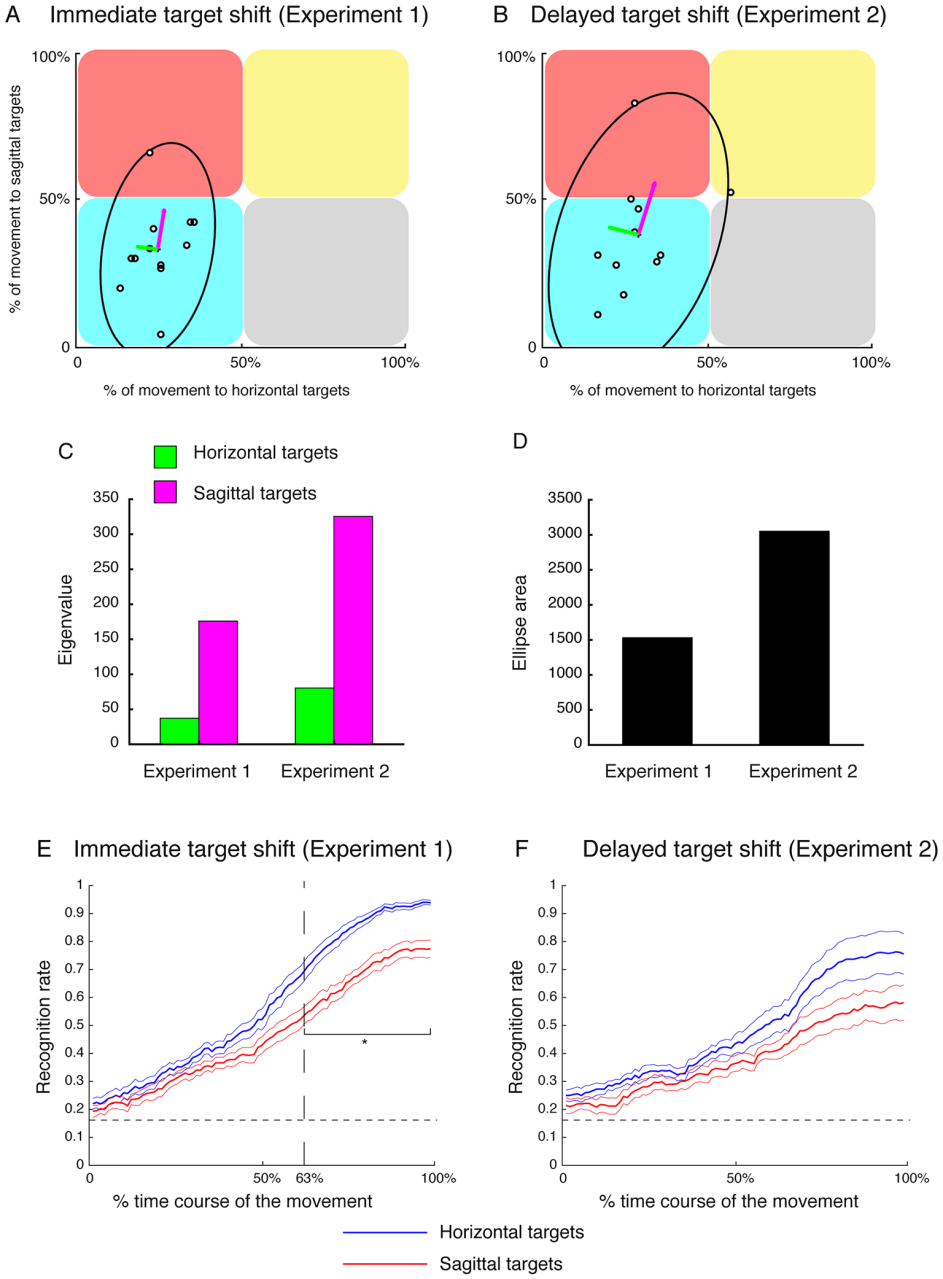
Analysis of the variability and accuracy in decoding horizontal and sagittal targets in the two experiments. (**A**) Average percentage of the movement where the classification accuracy exceeded 0.5 in decoding horizontal (x axis) and sagittal (y axis) targets in Experiment 1. Each point represents a participant. The confidence ellipse encircled the 95% of the data, the pink and green arrows represent eigenvalues from which the confidence ellipse was computed. Specifically, green and pink arrows represent the spread of the data in the direction of the eigenvectors for horizontal and sagittal targets, respectively. (**B**) Average percentage of the movement where the classification accuracy exceeded 0.5 in decoding horizontal (x axis) and sagittal (y axis) targets in Experiment 2. Details as in (**A**). (**C**) Histogram represents the eigenvalues for horizontal and sagittal target in the two Experiments. (**D**) Histogram represent the area of ellipses calculated in Experiment 1 and Experiment 2. (**E**) Average time course across participants of classification accuracy of static and shifted horizontal (blue line) and sagittal (red line) targets in Experiment 1. Blue and red thin lines are standard errors. The vertical dotted line represents the point of significant separation of the two lines assessed by Hotelling’s test (p < 0.05). The horizontal solid line represents the chance level (0.16). (**F**) Average time course across participants of classification accuracy of static and shifted horizontal (blue line) and sagittal (red line) targets in Experiment 2. Details as in (**E**).

The previous classification analysis enabled to assess the differences in the discriminability of static and shifted targets in the temporal domain. Apparently, no differences are visible from the time course of shifted target classification between the horizontal and sagittal dimension in Experiment 1 and 2. To further address this question, we first calculated at which time of the movement execution the recognition rate reached the chance level (0.5) in each participant when they must execute movement corrections towards targets along the horizontal and sagittal axes, respectively. Figure 7A shows the data of the chance level time for reaching to shifted sagittal targets as function of the chance level time for reaching to shifted horizontal targets in Experiment 1 at single subject level (individual white dots). We then constructed the confidence ellipse within which 95% of the points were located and computed the corresponding eigenvalues along the principal axes of the ellipse. In Experiment 1, the distribution of the points was mostly located in the left-bottom quadrant (blue square) suggesting that all participants reached the chance level in classification accuracy during the first half of the movement in both movements to shifted horizontal and sagittal targets. However, Fig. 7A, C (left) show that the magnitude of the eigenvalue corresponding to sagittal targets (pink vector and column) was larger than the eigenvalue corresponding to horizontal targets suggesting an increased variability in the classification of targets located along the sagittal dimension. Similar results were found in Experiment 2 where most of the participants reached the chance level of target classification within the half of the movement in both horizontal and sagittal targets (see Fig. 7B), but the increased variability in the classification of shifted sagittal targets was more pronounced with respect to Experiment 1 (Fig. 7B, C-right). The larger variability in target classification of Experiment 2 is evident also from the comparison of the two ellipse areas in Fig. 7D. These results suggest that the discrimination of static targets was significantly more accurate with respect to that of shifted target in both experiments and in both dimension of the space tested (horizontal and sagittal targets). Additionally, the classification of horizontal shifted targets resulted less variable and, consequently more stable, with respect to the classification of sagittal shifted targets. The stability of classification decreased with the increased latency of target shift as the variability results demonstrated in Fig. 7 (comparison between Experiment 1 and Experiment 2).

To further investigate the overall effect of horizontal and sagittal dimension not only in terms of variability in the predictability of final target goals, we calculated the averaged temporal evolution of the accuracy in recognizing static and shifted target positions along the two dimensions considered and in both experiments. As it is shown in Fig. 7E, we observed a progressive increase of the classification accuracy from the onset to the end of movement, well above the chance level, when the participants executed Experiment 1. However, classification accuracies in decoding targets along horizontal and sagittal dimensions showed differences in the maximum accuracy reached by the classifier in the final phase of the movement. In fact, for horizontal targets, the maximum accuracy was 0.94, whereas, for sagittal targets, the maximum accuracy was 0.77. Moreover, during the entire course of the movement, the classification accuracy of horizontal targets showed higher values with respect that of target in depth as it is shown in Fig. 7E, but this difference reached the significance level from the 63% (~ 396.18 ms after the movement onset) of the reaching execution (SPM1d with two-sample Hotellings’ T2 test, p < 0.05). In Experiment 2, the comparison between the average recognition rate across the movement execution displays that the prediction of static and shifted horizontal target goals was more accurate than the prediction of static and shifted sagittal target goals, consistently with the results of Experiment 1 (see Fig. 7F). However, no significant differences between decoding accuracy of horizontal and sagittal targets were found (SPM1d with two-sample Hotellings’ T2 test, p > 0.05). These results suggest that when the target was located along the sagittal dimension the target recognition was less accurate than the targets located along the horizontal dimension in both experiments. However, if the target shift occurred earlier (movement onset), the target recognition improved also along the sagittal dimension.

### Trajectory reconstruction of movements to static and shifted reaching targets located along the horizontal and sagittal dimension.

**Figure 8:**
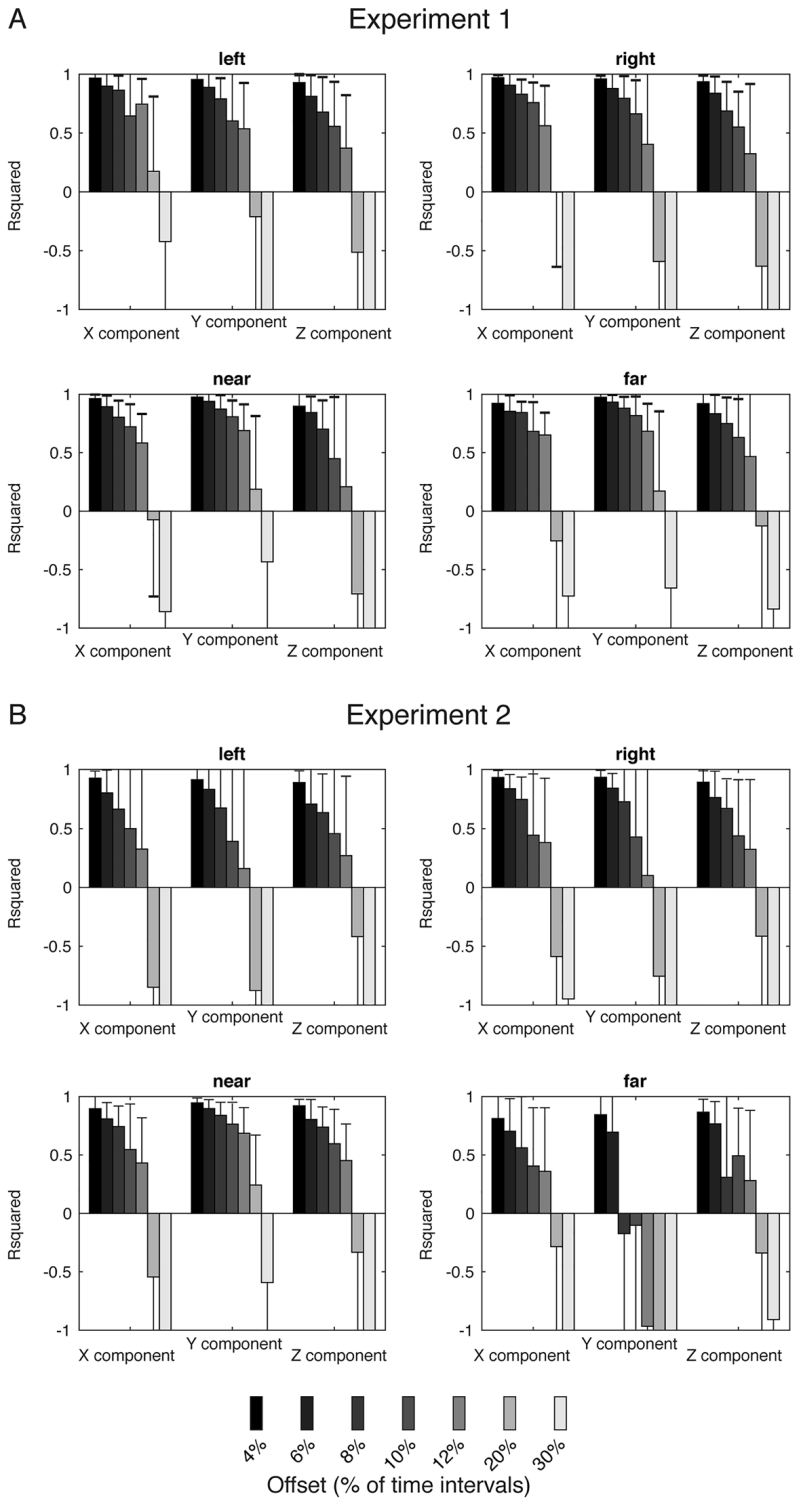
R squared distribution of decoded X, Y and Z trajectory component (height of bars: mean value, error bars: standard deviation) as scored by RNN regressors. (**A**) Left, right, near and far target clusters in Experiment 1. (**B**) Left, right, near and far target clusters in Experiment 2. In all panels, each target cluster includes the static and the two corresponding shifted targets. No differences between horizontal and sagittal dimension are present in the prediction accuracy of future kinematics.

To assess the ability in reconstructing the future trajectory observing the past one and how this was influenced by the offset between past and future observations, RNN-based regressors were trained on sliding windows within the trial course by varying the offset between past (regressor input) and future (regressor output) kinematic values. Figure 8 shows the R squared for the three trajectory components (x, y and z) averaged across the time course of the movement, respectively; these were computed for each sector of the tested space (left, right, near and far). In all the trajectory components and for all the tested target positions, it was evident a progressive decrease of the ability in reconstructing the future trajectory in the intervals lasting between 4 and 12% of movement duration from the past observations. In the attempt to predict the future trajectory samples with intervals lasting 20% and 30% of the total movement duration, the RNN performance was dramatically reduced. However, despite the observed and expected decrease as the offset between future and past observations increases, in most cases ≥ 70% of the variability in the data was explained by RNNs for offsets up to 8% of the movement, i.e., for predictions distant up to 8% of movement. Lastly, similar R squared distributions were found for targets located along direction and depth dimensions in the two performed experiments and in all the three trajectory components tested (two samples Kolmogorov–Smirnov test, p > 0.05). While the recognition of the endpoint to reach resulted differently affected by the spatial position of the targets, e.g., see Fig. 7 for the differences in classification accuracy found for targets arranged in the two dimensions of the space (horizontal and sagittal targets), the prediction of future position values during movement was similar for horizontal and sagittal dimensions across the time steps. Together with the results obtained in the previous section, this result corroborates the idea that, regardless of the timing of the shift of the target endpoint, intermediate kinematics is not influenced by the dimension in which the shift of the perturbation occurs (i.e., depth or direction), as quantified by the prediction of future kinematics during the reaching movement (e.g., prediction of kinematic values of the next 12% of movement), but only the final reaching goal is.

## Discussion

The focus of this study was to assess whether it was possible to predict static and shifted target goals in the three-dimensional space based on advanced information from the hand kinematics of individual participants. Specifically, we explored (i) at which stage of the movement execution it was possible to make the most accurate predictions about final target goals and (ii) the influence of the offset between past and future observations on the reconstruction of the future trajectory observing the past one.

We defined temporal and accuracy differences in predicting static and shifted targets when these were moved at the onset of the movement (Experiment 1) or 100 ms after the onset of the movement (Experiment 2) and when they were located along the horizontal or along the sagittal axis. We found that the accuracy in predicting static targets was higher than the accuracy in predicting shifted targets in both experiments, but an increased variability was visible in predicting shifted targets located along the sagittal axis, particularly, during the execution of Experiment 2.

In this study, we found a progressive increase of decoding accuracy of stable targets across the movement execution that goes beyond the chance level before the half (50%) of action execution in both experiments (see Figs. 5 and 6, black lines). In fact, before the half of the movement, it was possible to predict the final target of the reaching with accuracy of ~ 90% (recognition rate: 0.9). However, we observed a different progression of the decoding accuracy of shifted targets. When the target shift occurred at the onset of the movement, the highest decoding accuracy similar to that of static targets was reached on averaged around the 62% of the movement with similar values for horizontal and sagittal targets. When the target shift occurred after 100 ms the movement onset, the highest decoding accuracy, similar to that of static targets, was reached on averaged ~ 76.5% of the movement for horizontal targets and around the end of the movement (~ 96.8%) for sagittal targets. All these differences can be ascribed to the kinematic properties related to the reorganization of movements following a shift of the target location. We found that participants took between 100 and 200 ms to correct movements towards horizontal targets in both experiments whereas they took more than 200 ms to correct movements towards sagittal targets when the target shift occurred at the onset of the action. However, no significant corrections were detected in movements towards sagittal targets when the target shift occurred after 100 ms the movement onset (Experiment 2). The correction times related to movements towards horizontal targets are aligned with what Parblanc and Martin (1992)^15^, Soechting and Lacquaniti (1983)^16^ and Brenner and Smeets (1997)^17^ found. In fact, they found the same range of correction time (between 100 and 200 ms) to respond adequately to an unpredictable displacement of the reaching targets during the movement confirming that some corrections can be made during most movements. However, we found that movement corrections to sagittal targets were delayed or not detectable according to the time of the target displacement, respectively. The differences found for movements towards horizontal and sagittal targets are also evident in the target classification in terms of prediction variability of shifted targets and in terms of prediction accuracy of static and shifted targets together (Fig. 7). These results are in accordance with several behavioural studies demonstrating that depth and direction are two spatial parameters processed independently^18,19^. At neural level, it was shown that going from parietal to frontal areas, the processing of depth and direction information is more segregated in both humans and monkeys^20,21^. The decoding of target goals taking into account the depth dimension was mostly addressed in studies where the input data were represented by neural signals recorded from monkey^22^ and human^23,24^ brain areas and not by pure kinematics as in the present work (i.e. x, y and z components of hand trajectory). Although in monkey the results showed a reliable decoding of targets goals also in depth from parietal neural discharges^22^, in humans, when the depth information was added, the decoding of movement became reasonably slower and clumsier^23^. This can be explainable by the evidence that visual depth perception is distorted and the same binocular disparity can be generated by different objects^25–28^. For example, based on disparity information alone, a marble ball that you hold between the fingers and a basketball at a distance of two meters appear virtually identical. In theory, the brain can resolve this ambiguity from an estimate of fixation distance provided by the ocular convergence. However, the brain fails to properly calibrate relative depth, because perceived relative depth is overestimated for objects that are closer from the observer and underestimated for objects that are further^29^. A similar distortion can be observed also for pointing to visual targets in depth^30^, so reflecting the kinematics of these types of movements and the accuracy through which the final targets are predicted. However, if the depth distortion perception can explain the differential effect of direction and depth dimension on the classification accuracy of the final targets, the same cannot be considered to explain the absence of the same effect on the accuracy of intermediate kinematics prediction. In this case, similar accuracies were found in our results suggesting that depth and direction are relevant factors for predicting reaching execution as function of the final goals but not for predicting the reaching execution itself.

Another aspect of the present study is related to the higher classification accuracy found for the target shifts occurring at the movement onset (Experiment 1) with respect to those occurring after 100 ms after the movement onset (Experiment 2). These results are interesting in the framework of models of goal directed reaching, suggesting that targeted reaching movements require several processing steps^31^. The target is first localised, a motor plan is generated, and finally motor commands are sent to the arm muscles, resulting in a movement. During the movement process a forward model of the dynamics of the arm is generated. This model receives the sensory inflow and a copy of the motor outflow as inputs and generates an estimate of the movement endpoint location as output. Visual and proprioceptive feedback is used to compare this estimate with information about the target location. The difference between actual and predicted sensory feedback is the sensory error and in the case of discrepancy, an error signal is produced that triggers a modification of the ongoing motor command^31^. Through this online control process, sensory feedback and feedforward information can be used during the reach to influence the outcome^32^. In this framework, previous work has demonstrated that the timing of the target perturbation and the subsequent time available to complete the movement correction limit the final accuracy of the movement. In fact, in this study, the accuracy in pointing the final shifted goals is significantly smaller when the target shift occurred later with respect the movement onset than when the target shift occurred at the movement onset. These results are in line with the study of Komilis et al. (1993)^33^ that conducted a double-step reaching task where the shift occurred at either reach onset or peak velocity. They demonstrated that when the target was shifted at movement onset participants compensated for 88–100% of the target displacement. In contrast, when the target was displaced at peak velocity participants were unable to fully account for the change in target position and compensated for only 20–40% of the perturbation^33^. Liu and Todorov (2007)^34^ similarly found incomplete corrections to target shifts that occurred considerably after reach onset. They suggested that achieving stability in stopping a reaching movement may potentially compromise the sensorimotor system’s ability to respond to positional errors at the end of the reach^34^. Present results are in accordance with these pieces of evidence, suggesting that target shifts occurring early in the reach (i.e. 0 ms) were more easily tackled by the neural network than those occurring later in the reach (i.e. 100 ms post reach initiation).

## Conclusions

The results of this study provide novel insights into quantitative estimate of the accuracy that is possible to achieve when predicting, from hand kinematics, whether a reaching target goal is located along the horizontal dimension (direction) or the sagittal dimension (depth) of the 3-dimensional space and when the target shift occurs at the onset of the reaching or at later stages in the reach, as a function of time during movement. Furthermore, we also extended this analysis not only for decoding reaching goals but also for intermediate positions, to investigate whether the previous results held also when predicting intermediate kinematics and not only the final goal. We achieved this by exploiting the prediction of a learning system (here a RNN) that provided evidence of the kinematic cues potentially available for predicting final action goals and the intermediate kinematics in reaching targets in the 3D space. Future directions will consider the extension of this framework towards applications in human–robot interaction in which more complicated actions and composite contexts need to be decoded for fruitful human-artificial intelligent agents.

## Materials and methods

### Participants

A total of 23 naïve volunteers (11 males and 12 females, mean age 22.6 ± 2.3) took part at the study. 12 participants performed Experiment 1 and 11 participants performed Experiment 2. All participants were right-handed, and they had normal or corrected-to-normal vision with no history of neurological or psychiatric disorder. The study was approved by the local ethical committee of University of Bologna and was conducted according to the principles expressed in the Declaration of Helsinki. All participants provided written informed consent.

### Experimental setup

In all trials, the starting position of the hand (dominant right hand) was on a computer mouse placed adjacent to the touchscreen within a square marked with a tape (size 12 × 12 cm) in front of the participant’s chest, as sketched in Fig. 1A.

Reaching movements were performed in a dimly illuminated room. The head of participants was supported on a chinrest in order to reduce movements. The stimuli were green (diameter 0.3 cm) and red dots (diameter 1.2 cm) presented at different depths and at different direction with respect to the participant’s midline. The stimuli presented a luminance of ~ 17 cd/m^2^. Stimuli were presented on 19-inch touchscreen (ELO IntelliTouch 1939L) laid horizontally on a desk located at waist level with a visible display size of 37.5 × 30 cm and 15,500 touchpoints/cm^2^. The display had a resolution of 1152 × 864 pixels and a frame rate of 60 Hz.

Reaching movements were recorded using a motion tracking system (VICON motion capture system) by sampling the position of two markers at a frequency of 100 Hz; markers were attached to the wrist (on the scaphoid bone) and on the nail of the index finger (pointing finger). Participant’s eye positions were recorded by a mobile eye-tracking glasses (Pupil Core, Pupil Labs GmbH., Berlin, Germany). This device is composed of three cameras: a world camera which records the subject’s field of vision (resolution: 1920 × 1080 pixels; field of view: 100° fisheye; sampling rate: 60 Hz) and two eye-cameras that records the observer’s eye movements (resolution: 1920 × 1080 pixels; sampling rate: 120 Hz). Before each measurement, a binocular calibration process was carried out automatically using five pupil calibration markers (v0.4) located in the touchscreen’s corners and center. The psychophysical experiment was designed and generated with Matlab (Mathworks, USA) with the Psychophysics toolbox extension^35^. Arduino UNO microcontroller (Arduino UNO, Arduino, Milan, Italy) was responsible for triggering the start/stop of the VICON system capture.

### Behavioural task

The scheme of the experimental paradigm is illustrated in Fig. 1B. All participants performed a peripheral reaching task where the movement was executed maintaining the fixation on the central position of the screen. The experimental procedure consisted in the presentation of a central fixation point that prompted the participant to press the left mouse button (HB). Then, the participant had to stare at the fixation point for a period of 1.5 s. After this interval, the reaching target appeared (Cue/Go) indicating the position to reach and that the participant had to promptly reach that target position while maintaining fixation on the central fixation point. The end of trial was triggered to the screen touch. In the half of the trials, the reaching target could remain static in the same position (static trials) and in the remaining half of trials, the target shifted in another position (shifted trials) according to the target arrangement (see Fig. 1A). The static targets were presented at the distance of 8 cm with respect the fixation point and the shifted targets were presented at 2.4 cm with respect the initial static target in both horizontal and sagittal dimensions. Experiment 1 and Experiment 2 were identical except in the time of target position shift in shifted trials. In Experiment 1, it was triggered with movement onset (left mouse button release, see Fig. 1B, top) and, in Experiment 2, it was triggered 100 ms after the movement onset (see Fig. 1B, bottom). In the shifted trials, the participants had to suddenly, unexpectedly online correct the reaching movement for the unexpected perturbation, to touch the new target position that could be located farther or nearer than the previous, for targets located along the depth dimension (shift in depth, Fig. 1A) or rightward or leftward to it, for targets located along the direction dimension (shift in direction, Fig. 1A). Both the fixation point and the reaching target remained illuminated until the participant had completed the arm movement (visually guided reaching). The participant had to fixate the fixation point all throughout the trial. After touching the target, the target and the fixation point switched off and a new trial started. Participants were asked to move the hand in a ballistic way (without pauses or interruptions), at a fast but comfortable speed, and as accurately as possible. During an experimental session, each participant completed 120 trials (60 static trials and 60 shifted trials).

### Data processing and analysis

The analyses were performed with customized scripts in MATLAB (The MathWorks; RRID: SCR_001622) and Python 3 using open source machine learning (scikit-learn, http://scikit-learn.org, RRID: SCR_002577) and deep learning (Keras, https://keras.io/) Python packages. Specifically, for hand position data processing and analysis, a custom MATLAB algorithm was used to extract the x, y and z components of the index and wrist trajectories between the onset and the offset of the reaching movement (see Fig. 1C). For gaze data processing and analysis, the Pupil Player tool was used to extract gaze position data in normalized coordinates (see Fig. 1D). A custom MATLAB script was created to convert the normalized data into degree (deg), considering viewing distance and size and resolution of the Pupil Labs world camera. For data filtering, only data related to confidence values greater than 0.6 were used. The gaze position data was used only to check whether the position of the eyes was on the fixation point at the center of the screen during the reaching execution.

To evaluate the response to the target perturbation, we calculated the average lateral velocity for reaching movements performed towards left, right, near and far shifted targets (the velocity of the x component of the trajectory) during the entire execution of the movement and for each participant. Then, we compared the lateral velocities of left and right shifted trajectory and those of near and far shifted trajectory in each sample of all participants by a two-tailed t-test (p < 0.05). By this method, we found the sample at which the two shifted trajectory started to become statistically different (two-tailed t-test, p < 0.05).

Movement accuracy was extracted by endpoints recorded as the x and y coordinates of the touching point acquired by the touchscreen and the x and y coordinates of the target location. The measure of accuracy was extracted for each participant and calculated as follows:

$$Accuracy=\sqrt{{\left({x}_{M}-{x}_{loc}\right)}^{2}+{\left({y}_{M}-{y}_{loc}\right)}^{2}}$$

where ${x}_{M}$ and ${y}_{M}$ are the average endpoint coordinates and ${x}_{loc}$ and ${y}_{loc}$ are the target location coordinates. Then we averaged the values of accuracy across targets located along horizontal and sagittal axis and across all participants. We compared the accuracy between horizontal and sagittal within each experiment by a two-tailed t-test (p < 0.05).

Reaction times were calculated as the time interval between the appearance of the reaching target and the movement onset. We compared the reaction times between the two experiments by a two-tailed t-test corrected for multiple comparisons (p > 0.0125).

As measure of motor strategies along the movement execution, we performed an analysis of the variability of trajectories across trials. For each participant and in each experiment, we calculated standard deviations across trials in both X (for static and shifted horizontal targets) and Y dimensions (for static and shifted sagittal targets) at a relevant point of the movement corresponding to the peak velocity (point of maximum velocity); then we averaged across participants^36–38^.

### Analyses based on kinematics decoding

Due to the different number of samples of each trial (i.e., each trial lasted differently), position trajectories of index and wrist belonging to each trial were normalized between 0 and 100% of the trial duration and resampled to have a fixed number of 100 samples. The x, y, z positions of index and wrist movement effectors were analyzed using two different sliding window decoding approaches, decoding a single chunk of kinematic activity at a time. For both approaches, each input chunk consisted of the $K=6$ kinematic variables $\left[{p}_{x}^{index},{p}_{y}^{index},{p}_{z}^{index},{p}_{x}^{wrist},{p}_{y}^{wrist},{p}_{z}^{wrist}\right]$ evaluated at $\mathrm{T}=8$ consecutive time steps, i.e., the i-th extracted chunk ${X}_{i}$ was a 2D matrix with shape $(T,K)$, ${X}_{i}\in {\mathbb{R}}^{T\times K}$. That is, the input feature space included 6 dimensions corresponding to x, y, z of the index and of the wrist, and to better capture the dynamics of the system, each training example given to the network included 8 time steps. During network training, each input chunk slid with a stride of 4 time samples over the total number of 100 samples characterizing each trial, to reduce the computational cost. Then, each so extracted chunk ${X}_{i}$ was provided as input to the decoding stage. During the network inference step, input chunks slid with a unitary stride to increase the inference time resolution of the performed analyses. Due to the sliding window nature of decoding, the discriminatory capability (as quantified by the performance metrics) of learning systems can also be analyzed as a function of time, thus enabling the analysis of the predictability of reaching from kinematics in the temporal domain. This was performed in the approach (i) (see the following) in which we were interested into analyzing the temporal structure of decoding performance, while in the approach (ii) the analysis was conducted on average across time points.

In the following, the decoding analyses are described.

(i) *Classification of reaching goals*. In this first decoding analysis, central for this study, classifiers were trained to predict the target endpoint to reach, given the x, y, z coordinates of the index and wrist contained in the chunk ${X}_{i}$. The desired output was set to the class of the final target to which the whole trial corresponded. With this approach, we pointed to test when, during movement execution, it was possible to accurately detect the reaching goal from kinematics. Specifically, classifiers were trained and tested to discriminate between pairs of endpoints (2-class classification), separately for different latencies of the applied perturbation (i.e., different experiments conducted), and separately for targets located along horizontal and sagittal dimensions (see Fig. 1A for the endpoints location) and for static and shifted conditions. This last selection was provided by using the trajectory data of reaching for pairs of static targets or shifted targets in a specific dimension (depth or direction, see Fig. 1A). Overall, this decoding analysis was devoted to compare the different temporal dynamics of predictability of reaching goals at different levels, i.e., horizontal vs. sagittal dimensions, static vs. shifted movements, 0 ms vs. 100 ms perturbation latency respect to movement onset. For the direct comparison between classification of horizontal and sagittal targets reported in Fig. 7E, F, the classifiers were trained and tested to discriminate the 6 endpoints (6-way classification) separately for horizontal and sagittal dimensions.
  Furthermore, to inspect the differences in classification accuracy between horizontal and sagittal shifted targets in each experiment, we computed the averaged sample at which the accuracy exceeded the chance level (0.5) in recognition. Then, we plotted the percentage of movement towards shifted horizontal targets at which the classification accuracy exceeded the value of 0.5 against the percentage of movement towards shifted sagittal targets exceeded the same value. The differences between the classification accuracy were evaluated by calculating the confidence ellipses for 2D normally distributed data in the two experiments separately. The shape of the confidence ellipses was determined by computing the eigenvectors and the eigenvalues. Eigenvectors represent the direction in which the data varies the most and the eigenvalues correspond to the spread of the data in the direction of the eigenvectors. The area of the ellipses was computed from the length of the major and minor axes defined as standard deviations σ_x_ and σ_y_ of the data.

(ii) *Regression of future trajectory values*. In this analysis, we analyzed to which extent the intermediate kinematic observations (and not only the final reaching goal) were predictable from past kinematics. Regressors were trained to predict the future position trajectory values recorded during movement, given the x, y, z coordinates of the index and wrist contained in ${X}_{i}$. Remarkably, trajectory regression was performed as a function of the interval (hereafter referred as “offset”) between past and future observations; future observations were sampled with an offset of 4, 6, 8, 10, 12, 20, 30 time samples with respect to past observations. As each trial was normalized between 0 and 100% of the performed movement and resampled to 100 time steps, the investigated offsets corresponded to infer observations in the future at 4%-30% of movement from past observations. This analysis was conducted separately for different latencies of the applied shift (i.e., different experiments conducted) and separately for near, far, left and right targets (thus, separately for targets located on horizontal vs. sagittal dimensions), by including in each of these cases the trajectory data of reaching towards the static target and the two corresponding shifted targets (see Fig. 1A).

Regarding network structure, RNNs were used to address both decoding problems. RNN-based classifiers and regressors shared the same input and hidden layers. Specifically, an input layer simply replicating the input chunk ${X}_{i}$ was used, and a single hidden layer based on Gated Recurrent Units (GRUs)^39^ with 100 GRU units was designed. The networks changed only in the adopted output layer across the two decoding approaches. Therefore, in the classification task the output layer was a dense fully-connected layer with one unit for each reaching endpoint, that is, 2 units (discrimination among pairs of targets, separately within depth and direction dimensions), followed by a softmax activation function. Conversely, in the regression task, the output layer was a dense fully-connected layer with one unit for each trajectory component, that is, 6 units (corresponding to $K$).

Furthermore, as concerning network training, RNNs were trained using the categorical cross-entropy and mean squared error as loss functions for the addressed classification and regression tasks, respectively. Adam was used as optimizer^40^, with a learning rate of 0.001 and a batch size of 32. Network trainings were performed up to 10,000 epochs but were early stopped when the loss evaluated on a held-out validation set (set as 20% of the training set) did not decrease after 500 epochs. Unless not otherwise specified, the default Keras parameters were used (e.g., to define the network layers).

Lastly, regarding performance evaluation we adopted a fivefold stratified cross-validation scheme, thus, 5 independent models were trained using 48 training trials and 12 test trials within each fold, balanced across classes. Then, performance metrics (accuracy for classification and R squared for regression) scored on the test set were computed and averaged across the 5 cross-validation folds.

## Data Availability

The data that support the findings of this study are available from the corresponding author upon reasonable request.
